# A case report of rhabdomyolysis and osteofascial compartment syndrome in a patient with hypothyroidism and diabetes

**DOI:** 10.1186/s12902-021-00868-6

**Published:** 2021-10-24

**Authors:** Lijue Ren, Cuiying Wei, Feng Wei, Ruiting Ma, Yan Liu, Yonghong Zhang, Wei Wang, Jing Du, Lin Bai, Yexia Xue, Shaohua Cui

**Affiliations:** 1 Department of Endocrinology, First Affiliated Hospital of Baotou Medical College, Inner Mongolia University of Science and Technology, 41 Linyin Road, Kun District, Baotou City, 014010 Inner Mongolia China; 2 Intensive Care Unit, First Affiliated Hospital of Baotou Medical College, Inner Mongolia University of Science and Technology, Baotou, 014010 Inner Mongolia China

**Keywords:** Hypothyroidism, Rhabdomyolysis, Osteofascial compartment syndrome, Foot drop, Case report

## Abstract

**Background:**

Hypothyroidism is frequent and has various forms of muscle involvement. We report the diagnosis and treatment of a case of rhabdomyolysis, bilateral osteofascial compartment syndrome (OCS) of the lower extremities, and peroneal nerve injury causing bilateral foot drop in a diabetic patient with hypothyroidism.

**Case presentation:**

A 66-year-old man with diabetes for 22 years was admitted because of drowsiness, tiredness, facial swelling, and limb twitching for 2 months, and red and swollen lower limb skin for 3 days. Serum creatinine kinase (CK), CK-MB, myoglobin (Mb), blood glucose, and HbA1c were elevated. TSH, thyroid peroxidase antibodies, and antithyroglobulin antibodies were elevated. FT3 and FT4 were low. Urine was dark brown. He was diagnosed with hypothyroidism, rhabdomyolysis, and OCS. CK, CK-MB, and Mb returned to normal after treatment with thyroid hormone, insulin, albumin infusion, ceftriaxone, ulinastatin, and hemofiltration, and the redness and swelling of the lower limbs were relieved, but the patient developed dropping feet. The patient recovered well but had to undergo rehabilitation.

**Conclusion:**

Hypothyroidism may induce rhabdomyolysis, OCS, and other complications. This case reminds us of the importance of screening for hypothyroidism and strengthens the clinicians’ understanding of the disease.

**Supplementary Information:**

The online version contains supplementary material available at 10.1186/s12902-021-00868-6.

## Background

Hypothyroidism is a frequent occurrence in the endocrinology department, with a prevalence of 1–2% in iodine-sufficient countries but can reach 5.3% in some European countries [1]. The common clinical manifestations are systemic metabolic reduction syndromes such as cold sensitivity, fatigue, memory loss, and edema [2, 3]. About 79% of adult patients with hypothyroidism have various forms of muscle involvement such as myalgia, muscle cramps, and muscle stiffness [4].

Rhabdomyolysis (RM) and osteofascial compartment syndrome (OCS) [5, 6] are rare manifestations of hypothyroidism. RM can cause acute kidney injury (AKI), electrolyte disturbance, metabolic acidosis, and OCS in the presence of limb compression, leading to morbidity and even mortality. Hypothyroidism, combined with the above complications, is extremely dangerous and needs timely treatment [2, 3, 7–9].

We report the diagnosis and treatment of a case of RM, bilateral lower extremity OCS, and peroneal nerve injury causing bilateral foot drop in a diabetic patient with hypothyroidism.

## Case presentation

A 66-year-old retired man (Han Chinese) was referred to the endocrinology unit of the First Affiliated Hospital of Baotou Medical College, Inner Mongolia University of Science and Technology, due to poor plasma glucose control and severely painful lower limbs on April 21, 2020. He reported sleepiness, fatigue, swelling of the face, and occasional lower limb convulsions for the past 2 months. He complained of pain, redness, and swelling in the lower limbs for 3 days and gradually worsening symptoms, which severely affected his daily activities and sleep quality. The patient had a history of type 2 diabetes for 22 years. Due to the poor effect of metformin, he had been injecting mixed recombinant human insulin (30/70) 44 U/day for the past 5 years. His fasting blood glucose fluctuated around 9.0 mmol/L. The patient reported no history of trauma, excessive exercise, fever, drinking alcohol, and medication (other than insulin) in the past 6 months. The patient reported no family genetic history. Physical examination on admission showed stable vitals, with a heart rate of 72 bpm and blood pressure of 130/70 mmHg, but dry and pale skin and slow speech. The whole body was swollen, especially the lower limbs, and the anterior shin of both legs were red and swollen. Local skin tension was high, and tenderness was obvious.

The blood investigations revealed serum creatine kinase (CK) at 9774 U/L (reference: 50–310 U/L), CK isoenzyme (CK-MB) at 115.2 U/L (reference: 0–24 U/L), cardiac troponinI (cTnI) at 0 U/L, myoglobin (Mb) at > 3811 μg/L (reference: 0–70 μg/L), albumin (Alb) at 48.8 g/L (reference: 40–55 g/L), alanine aminotransferase (ALT) at 46 U/L (reference: 9–50 U/L), aspartate aminotransferase (AST) at 139 U/L (reference: 15–40 U/L), lactate dehydrogenase (LDH) at 579 U/L (reference: 120–250 U/L), and α-hydroxybutyrate dehydrogenase (HBDH) at 419 U/L (reference:72–182 U/L), suggesting RM. Blood glucose was 13.7 mmol/L (reference: 3.9–7.7 mmol/L), HbA1c was 10.6% (reference: 3.9–6.2%), hemoglobin was 159 g/L (reference: 130–175 g/L), white cell counts was 11.98 × 10^9^/L (reference: 3.5–9.5 × 10^9^/L), and platelet count was in the normal range (125–350 × 10^9^/L). Free triiodothyronine (FT3) was 0.06 pg/ml (reference: 2.3–4.2 pmol/L), free thyroxine (FT4) was 2.78 pmol/L (reference: 7.5–17.4 pmol/L), and TSH was 145.6 mIU/L (reference: 0.35–5.5 mIU/L). Subsequent tests were suggestive of Hashimoto’s thyroiditis, with raised thyroid peroxidase antibodies at 661.8 IU/ml (reference: 0–34 IU/ml) and positive antithyroglobulin antibody at 366.20 KIU/L (reference:0–115 KIU/L). Blood electrolytes, renal function, coagulation function, brain natriuretic peptide, chest radiograph, and abdominal ultrasound were normal. There were no erythrocytes on microscopic examination, although his urine was bloody in appearance. Ultrasound of both lower extremities suggested uneven thickening of the arteries and media of the lower extremities with plaques and no abnormalities in the veins of both lower extremities. Swelling of the lower limbs caused by vascular occlusion and thrombosis was excluded. There was no ST-T segment change on the ECG.

Considering RM, fluid replacement, maintenance of water and electrolyte balance, diuresis, alkalized urine, penicillin, insulin, thyroid hormone replacement, and loxoprofen for pain relief were given immediately. The lower extremity swelling continued to progress, with high tension and thin skin. Tension blisters appeared on the right ankle on April 22, and muscle enzymes increased progressively, urine was dark brown, urine output was 3500 ml/day. On April 23, creatinine did not elevate [116 μmol/L (reference: 57–111)], but CK rose at 48,118 U/L (reference: 50–310 U/L). Mb was > 3811 μg/L, ALT was 196 U/L, AST was 1027 U/L, LDH was 1422 U/L, and HBDH was 798 U/L. An orthopedist was consulted and suggested lower limb OCS. Surgery could not be considered due to the high risk of open decompression and complications such as poorly healing incision because of poor blood glucose control and hypothyroidism.

Hemofiltration was started on the evening of April 23. The lower limb pain and redness gradually reduced, and the levels of CK, CK-MB, Mb, ALT, AST, LDH, and HBDH gradually decreased. CK fell to 8301 U/L (reference: 50–310 U/L) and Mb to 1183 μg/L on April 28. Blood Alb and hemoglobin gradually decreased, and APTT and PT were prolonged. On April 29, there were large ecchymoses on the right waist and right thigh, and the anterior tibial swelling of the two lower legs was aggravated again. Ultrasound showed intermuscular hematoma of both lower legs and subcutaneous hematoma of the right thigh. The feet were drooping and could not stretch back. The worsening of the disease was considered related to abnormal blood coagulation caused by RM.

**Fig. 1:**
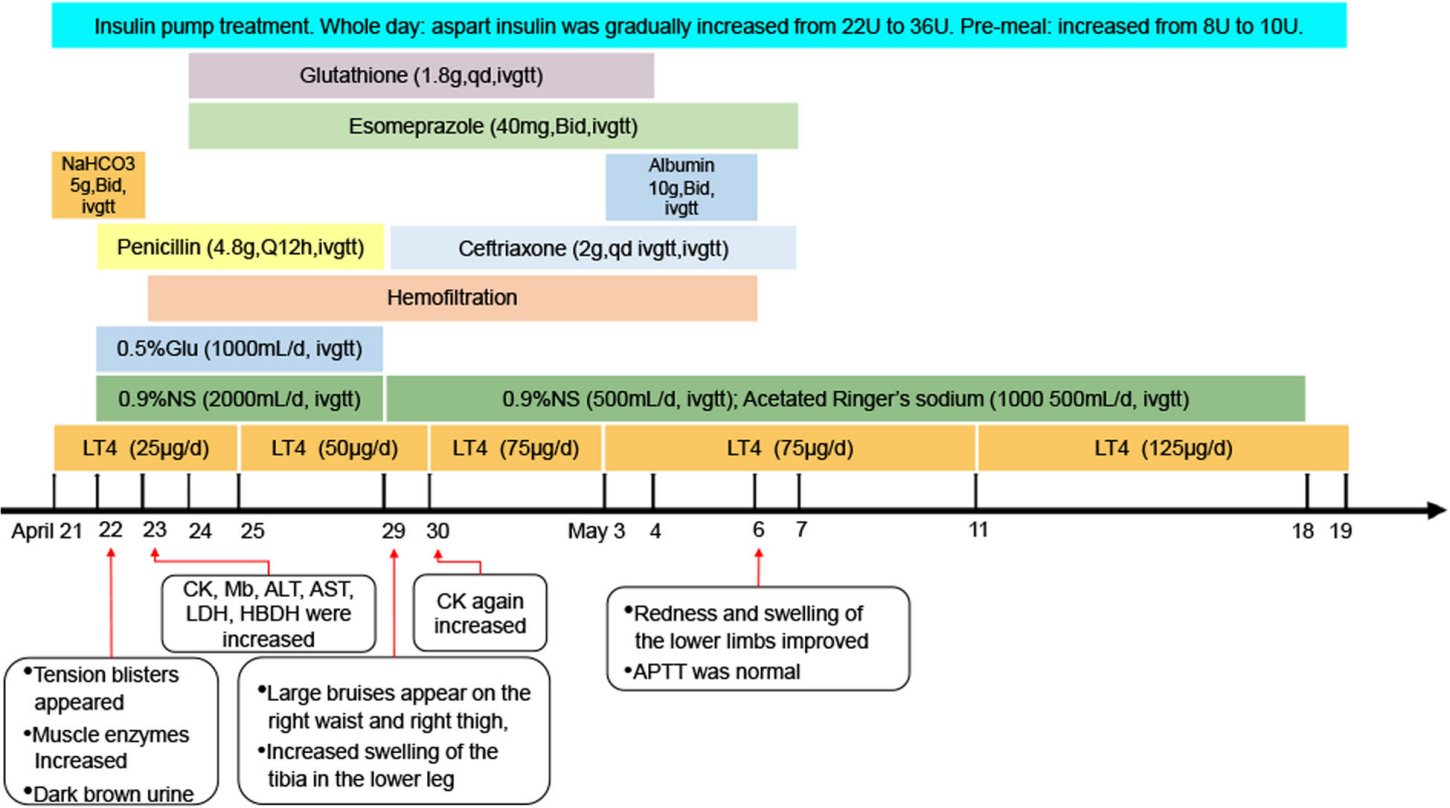
The medication used during the treatment of the patient. An insulin pump was used to control the blood glucose levels from admission to discharge. At admission, the basal amount of insulin asparagine was 22 U, and the dose before the three meals was 8 U. The insulin dosage was adjusted according to the blood glucose levels every 1–2 days, by increments of 2–4 U. After discharge, the patient was given hypodermic injection of insulin glargine 36 U before going to bed and insulin asparagine 10 U before the three meals. NS: normal saline; Glu: glucose; LT4: levothyroxine

Considering the necrosis of the soft tissue of the anterior tibia, ceftriaxone (2 g/qd) was given from April 29 to May 7. On April 30, the levels of CK again increased to 14,292 U/L. Blood filtration, fluid replacement, gradually increasing the dose of thyroid hormone (75 μg/d) and insulin (insulin pump therapy, basic dose 30 U, high dose 10 U before meals), infusion of albumin (20 g/day, continued for 1 week), and other treatments (glutathione 1.8 g/day and esomeprazole 40 mg/12 h) were continued. The condition of the patient gradually stabilized. On May 6, hemofiltration was stopped due to the improvement of the redness and swelling of the lower limbs, and APTT was normal. Since the levels of CK (4650 U/L) and Mb (232.0 μg/L) were still higher than the normal range, treatment was continued. Afterward, the lower limbs were slightly red but without pain and swelling. Muscle enzymes continued to decline, with CK from 4650 U/L to 1547 U/L and Mb to normal. FT3/FT4 gradually rose. Fasting blood glucose fluctuated around 7.0 mmol/L, and postprandial blood glucose fluctuated around 10.0 mmol/L. The foot drop did not recover (Fig. 1).

The patient was discharged on May 19. The lower limbs were slightly red but without pain and discomfort. CK was 1547 U/L, and Mb was normal. CK was checked every 10 days and FT3/FT4 every month after that. Rehabilitation was recommended. On May 30, the patient was able to stand, but walking was slightly unstable. CK was 818 U/L, and Mb was normal. On July 12, the patient’s feet were still drooping; there was no redness or swelling in the lower limbs and occasional swelling of the ankles. The levels of TSH, FT3, FT4, and CK were normal. The patient is undergoing rehabilitation. The patient’s treatment process is shown in Fig. 1. The changes in related laboratory indicators are shown in Supplementary Tables S1, S2, and S3.

## Discussion and conclusions

The causes of RM are diverse and can be generally divided into physical factors such as crushing, trauma, strenuous exercise, electric shock, and high fever, and non-medical factors such as drugs, poisons, infections, electrolyte disturbances, autoimmune diseases, and endocrine and genetic metabolic diseases [10]. In patients with hypothyroidism, CK can be slightly to moderately elevated [5]. Still, RM caused by hypothyroidism is relatively rare [5, 6], and the reported cases are mostly due to the use of lipid-lowering drugs such as statins or vigorous exercise [11, 12]. Salehi et al. [6] summarized 10 previously reported cases of RM caused by hypothyroidism in adults. One case was a patient with subclinical hyperthyroidism who was taking propylthiouracil. There were four patients with a known diagnosis of HS. In five patients, thyroid disease was not previously diagnosed. There were six cases without any risk factors for rhabdomyolysis.

The patient reported here had severe hypothyroidism, prominent symptoms of myalgia and fatigue, dark urine, serum CK up to 48,118 U/L, and Mb > 3811 μg/L, leading to a definite diagnosis of RM. The patient had no history of taking medication in the past 6 months, and there were no other known causes of RM, such as poison, exercise, and infection. Therefore, RM in this patient was considered to be caused by hypothyroidism. Furthermore, his antibodies to TPOAb and TgAb were increased significantly, indicating Hashimoto’s thyroiditis, which has also been reported as a possible cause of RM [13]. In addition, the incidence of adult RM in the diabetic state is about 10%, and the reported cases were all caused by acute complications such as diabetic ketoacidosis and diabetic hyperosmolar state [14, 15]. Severe dehydration, insufficient oxygen supply to the local tissues, and exhausted sodium and potassium pumps on the muscle cell membrane will lead to muscle cell lysis. At the same time, severe hyperglycemia activates aldose reductase in skeletal muscle cells, leading to the accumulation of large amounts of sorbitol and fructose, causing osmotic cell swelling and damaging skeletal muscle cells. Acute and chronic hyperglycemia can also produce various inflammatory factors that directly damage local muscle tissue [16, 17]. In the case reported here, the patient’s blood glucose levels were increased without predisposing factors such as hypernatremia, hypokalemia, and hyperosmolar state. Blood glucose at admission was 13.5 mmol/L. After insulin injection, the fast blood glucose was around 7 mmol/L, and postprandial blood glucose was around 10 mmol/L. Therefore, blood sugar might be involved in the occurrence of RM, but it is not the main cause.

The mechanism by which hypothyroidism causes RM is unclear. It is well known that the active form of thyroid hormone is triiodothyronine (T3), which plays a central role in muscle function and integrity. The main fuel substrate for muscle energy metabolism is glycogen. T3 can stimulate the metabolism of carbohydrates and increase the mobilization and utilization of glycogen in muscle tissue. Muscle contraction and relaxation mainly depend on the energy produced by adenosine triphosphate (ATP) hydrolysis and mitochondrial oxidative respiratory chain. T3 can regulate the above link [18, 19]. T3 also affects myosin thick filaments and muscles. Therefore, the currently accepted hypothesis of hypothyroidism leading to RM is that hypothyroidism inhibits glycogen breakdown and damages mitochondrial activity, causing a series of metabolic dysfunctions [20–22]. Hypothyroidism can convert muscle fibers from fast convulsions type II to slow convulsions type I so that actin-myosin has poor contractility, myosin ATPase activity is low, and ATP conversion rate in skeletal muscle is low, resulting in insufficient muscle blood vessel perfusion, hypoxia of muscle tissue, and low muscle energy storage [23].

OCS can be caused by reducing the size of the compartment or increasing its contents. OCS is usually unilateral and is found bilaterally in only < 10% of the cases [24]. Because the calf compartment is small and tight, any swelling inside it can easily cause OCS. OCS, as a complication of RM secondary to hypothyroidism, is a rare condition. In 1993, Thacker et al. [25] reported OCS with hypothyroidism for the first time. In 2015, Chaudhary et al. [26] mentioned that there were only five previous cases. In hypothyroidism, due to the proliferation of connective tissue in mucinous edema, the size of the bone fascia compartment might be reduced, and, at the same time, the increase in the content of the anterior tibia area might be due to interstitial edema, muscle necrosis, and swelling caused by glycosaminoglycan deposition [25, 27]. Increased capillary permeability, slow lymphatic drainage, and extravasation of interstitial protein-rich liquid can also participate in OCS [28]. The development of OCS in the calf can cause the compression of the common peroneal nerve and cause dropping of the feet or the deposition of glycosaminoglycans in the nerve sheath of the deep peroneal nerve might also cause the same effect [29].

The differences in the treatment of hypothyroidism might be related to the patient’s age, comorbidities, and the severity of muscle damage. Chaudhary et al. [26] described a case of hypothyroidism-caused rhabdomyolysis and acute OCS resulting in acute episodes of prolapsed feet. Although normal thyroid function was achieved in the case presented here, muscle weakness symptoms did not improve. The treatment of RM and OCS includes a combination of multiple measures. The treatment principle of RM is to remove the cause as soon as possible and give a large amount of fluid and alkaline urine treatment as early as possible to prevent and treat critical complications such as acute kidney injury (AKI). Continuous renal replacement therapy (CRRT) is an important and very effective treatment. If blood creatinine rises to three times the baseline value or reaches 353.6 μmol/L, urine volume is less than 0.3 mL/kg/h, or the patient is 12 h without urine, blood purification treatment should be started [30]. We believe that AKI has not occurred in this patient, and the electrolyte and creatinine were normal throughout the treatment process. The main reason is that CRRT was started within a short time after admission. Then, blood myoglobin, CK, and CK-MB decreased significantly, indicating that CRRT could effectively remove blood creatinine, myoglobin, interferon-α, interleukin (IL)-1, IL-2, and other substances, and preventing AKI, disseminated intravascular coagulation, and multiple organ dysfunction [31].

For OCS, especially traumatic OCS, surgical incision decompression can achieve good results. Nevertheless, it has been reported that the surgical decompression gains in hypothyroidism-related OCS are not large, and conservative treatment has similar results [32]. Since the patient reported here had a high surgical risk due to hyperglycemia and mucinous edema of hypothyroidism, surgery was not performed.

Clinicians should be aware that hypothyroidism can cause RM and OCS and can happen without predisposing factors. Therefore, it is important to check thyroid function in patients with RM. For patients with hypothyroidism combined with RM, early implementation of CRRT can effectively prevent AKI and multiple organ dysfunction. In addition, patients with increased muscle pain, obvious limb swelling, and increased muscle enzymes need to be screened for during treatment. This case reminds us of the importance of screening for hypothyroidism and strengthens the clinicians’ understanding of the disease.

## Supplementary Information

**Additional file 1: Supplementary Table S1.** Key biochemical values of patients during hospitalization and out-of-hospital. **Supplementary Table S2.** Thyroid function of patients during hospitalization and out-of-hospital. **Supplementary Table S3.** Coagulation function of patients during hospitalization.

## Data Availability

The datasets used and/or analyzed during the current study are available from the corresponding author on reasonable request.

## Abbreviations

RM: Rhabdomyolysis
OCS: Osteofascial compartment syndrome
AKI: Acute kidney injury
CK: Creatine kinase
CK-MB: creatine kinase isoenzyme
cTnI: cardiac troponinI
Mb: Myoglobin
Alb: Albumin
ALT: Alanine aminotransferase
AST: Aspartate aminotransferase
LDH: Lactate dehydrogenase
HBDH: α-hydroxybutyrate dehydrogenase
FT3: Free triiodothyronine
FT4: Free thyroxine
ATP: Adenosine triphosphate
