# Supplementary material for: A case report of rhabdomyolysis and osteofascial compartment syndrome in a patient with hypothyroidism and diabetes
Source: BMC Endocr Disord. 2021 Oct 24;21:212. doi: 10.1186/s12902-021-00868-6 (PMC8543899; doi:10.1186/s12902-021-00868-6)
Supplement: Supplementary file 1 — Additional file 1: Supplementary Table S1. Key biochemical values of patients during hospitalization and out-of-hospital. Supplementary Table S2. Thyroid function of patients during hospitalization and out-of-hospital. Supplementary Table S3. Coagulation function of patients during hospitalization. [file 12902_2021_868_MOESM1_ESM.doc]

**Supplementary Table S1.** Key biochemical values of patients during hospitalization and out-of-hospital.

| Time | CK  (U/L) | CK-MB  (U/L) | ALT  (U/L) | AST  (U/L) | ALB  (g/L) | Mb  (ug/L) | LDH  (U/L) | HBDH  (U/L) |
| --- | --- | --- | --- | --- | --- | --- | --- | --- |
| Reference | 50-310 | 0-24 | 9-50 | 15-40 | 40-55 | 0-70 | 120-250 | 72-182 |
| 21-Apr | 9774 | 115.2 | 46 | 139 | 48.8 | >3811 | 579 | 419 |
| 22-Apr | 36609 | 342.7 | 413 | 582 | 43.2 | >3811 | 1112 | 643 |
| 23-Apr | 48118 | 448.2 | 196 | 1027 | 40.2 | >3811 | 1422 | 798 |
| 24-Apr | 40583 | 393.8 | 212 | 949 | 38.8 | 3820 | 1311 | 777 |
| 26-Apr | 27466 | 245.6 | 201 | 828 | 38.2 | 3727.8 | 1046 | 682 |
| 28-Apr | 8301 | 189.5 | 174 | 615 | 32.9 | 1183 | 1028 | 728 |
| 30-Apr | 14292 | 144.1 | 163 | 431 | 35.6 | 920.4 | 941 | 690 |
| 1-May | 11464 | 119.9 | 139 | 328 | 33.7 | 623 | 831 | 618 |
| 3-May | 7284 | 87.3 | 110 | 236 | 33.5 | 419.3 | 670 | 544 |
| 6-May | 4650 | 50.9 | 51 | 103 | 34.2 | 232 | 410 | 320 |
| 9-May | 2839 | 44.6 | 40 | 89 | 33.3 | 143.5 | 487 | 299 |
| 13-May | 2328 | 39.2 | 37 | 90 | 37.3 | 110.3 | 392 | 299 |
| 16-May | 1547 | 31 | 35 | 77 | 35.6 | 59.2 | 339 | 285 |
| 19-May | 1602 | 31.3 | 34 | 67 | 38 | 46 | 330 | 281 |
| 30-May | 818 | 18.9 | 16.7 | 27.6 | 41.3 | 40 | 198 | 281 |
| 9-June | 453 | 14 | 18 | 29 | 45 | 28 | 188 | 162 |
| 12-July | 122 | 12 | 22 | 27 | 46 | 30 | 166 | 156 |

CK: creatinine kinase; CK-MB: creatinine kinase isoenzyme; ALT: alanine transaminase; AST: aspartate transaminase; ALB: albumin; Mb: myoglobin; LDH: lactate dehydrogenase; HBDH: α-hydroxybutyrate dehydrogenase.

**Supplementary Table S2**. Thyroid function of patients during hospitalization and out-of-hospital.

| Time | TSH  (mIU/L) | FT3  (pg/ml) | FT4  (pmol/L) | TgAb  (KIU/L) | TPOAb  (IU/ml) |
| --- | --- | --- | --- | --- | --- |
| Reference | 0.25-5.5 | 2.3-4.2 | 7.5-17.4 | 0.115 | 0-34 |
| 21-Apr | 145.6 | 0.06 | 2.78 | 366.2 | 661.8 |
| 11-May | >150 | 1.96 | 4.52 | - | - |
| 11-June | 22.57 | 2.96 | 16.48 | - | - |
| 12-July | 3.2 | 3.35 | 15.47 | - | - |

TSH: thyroid-stimulating hormone; TgAb: thyroglobulin antibodies; TPOAb: thyroid peroxidase antibody.

**Supplementary Table S3.** Coagulation function of patients during hospitalization.

| Time | PT-S  (s) | APTT  (s) | TT  (s) | FIB  (g/L) | D-D  (ug/ml) | FDP  (ug/ml) |
| --- | --- | --- | --- | --- | --- | --- |
| Reference | 8.8-12.8 | 24.9-40 | 11-18 | 2-4 | 0-0.5 | 0-5 |
| 21-Apr | 12.7 | 35.2 | 15.9 | 3.12 | 0.15 | 4.0 |
| 28-Apr | 13.9 | 62.3 | 17.6 | 4.32 | 0.41 | 2.36 |
| 29-Apr | 13.0 | 48.4 | 16.2 | 4.5 | 0.59 | 3.5 |
| 6-May | 13.0 | 36.4 | 15.4 | 3.11 | 0.49 | 4.01 |
| 13-May | 11.7 | 31.3 | 13.7 | 3.26 | 0.49 | 4.32 |

PT-S: prothrombin time; APTT; activated partial thromboplastin time; TT: thrombin time; FIB: fibrinogen; D-D: D-dimer; FDP: fibrin degradation product.
